# Evaluation of Multisite Programmatic Bundle to Reduce Unnecessary Antibiotic Prescribing for Respiratory Infections: A Retrospective Cohort Study

**DOI:** 10.1093/ofid/ofad585

**Published:** 2023-11-21

**Authors:** Dan Ilges, Kelsey Jensen, Evan Draper, Ross Dierkhising, Kimberly A Prigge, Paschalis Vergidis, Abinash Virk, Ryan W Stevens

**Affiliations:** Department of Pharmacy Services, Mayo Clinic Arizona, Phoenix, Arizona, USA; Department of Pharmacy Services, Mayo Clinic Health System–Southeast Minnesota, Austin, Minnesota, USA; Department of Pharmacy Services, Mayo Clinic, Rochester, Minnesota, USA; Division of Clinical Trials and Biostatistics, Mayo Clinic, Rochester, Minnesota, USA; Division of Family Medicine, Mayo Clinic, Rochester, Minnesota, USA; Division of Public Health, Infectious Diseases, and Occupational Medicine, Mayo Clinic, Rochester, Minnesota, USA; Division of Public Health, Infectious Diseases, and Occupational Medicine, Mayo Clinic, Rochester, Minnesota, USA; Department of Pharmacy Services, Mayo Clinic Health System–Southeast Minnesota, Austin, Minnesota, USA

## Abstract

**Background:**

The aim of this study was to evaluate the frequency of unnecessary antibiotic prescribing for Tier 3 upper respiratory infection (URI) syndromes across the Mayo Clinic Enterprise before and after a multifaceted antimicrobial stewardship intervention, and to determine ongoing factors associated with antibiotic prescribing and repeat respiratory healthcare contact in the postintervention period.

**Methods:**

This was a quasi-experimental, pre/post, retrospective cohort study from 1 January 2019 through 31 December 2022, with 12-month washout during implementation from 1 July 2020 through 30 June 2021. All outpatient encounters, adult and pediatric, from primary care, urgent care, and emergency medicine specialties with a Tier 3 URI diagnosis were included. The intervention was a multifaceted outpatient antibiotic stewardship bundle. The primary outcome was the rate of antibiotic prescribing in Tier 3 encounters. Secondary outcomes included 14-day repeat healthcare contact for respiratory indications and factors associated with persistent unnecessary prescribing.

**Results:**

A total of 165 658 Tier 3 encounters, 96 125 in the preintervention and 69 533 in the postintervention period, were included. Following intervention, the prescribing rate for Tier 3 encounters decreased from 21.7% to 11.2% (*P* < .001). Repeat 14-day respiratory healthcare contact in the no antibiotic group was lower postintervention (9.9.% vs 9.4%; *P* = .004). Multivariable models indicated that increasing patient age, Charlson comorbidity index, and primary diagnosis selected were the most important factors associated with persistent unnecessary antibiotic prescribing.

**Conclusions:**

Outpatient antibiotic stewardship initiatives can reduce unnecessary antibiotic prescribing for Tier 3 URIs without increasing repeat respiratory healthcare contact. Advancing age and number of comorbidities remain risk factors for persistent unnecessary antibiotic prescribing.

Approximately 80%–90% of all antimicrobial consumption occurs in outpatient settings [1]; however, up to 50% of these prescriptions may be inappropriate, with roughly 1 in 3 completely unnecessary [1–4]. Indiscriminate antibiotic prescribing is associated with harm to both individual patients (eg, adverse effects, such as diarrhea, rash, and photosensitivity) [5] and the broader population (eg, antimicrobial resistance and increased healthcare costs) [6]. In 2016, the Centers for Disease Control and Prevention published the Core Elements of Outpatient Antibiotic Stewardship, which encourages outpatient antimicrobial stewardship programs (ASP) to identify 1 or more high-priority targets [7].

Upper respiratory infections (URIs) represent the most common indication for outpatient antibiotic prescribing; however, a majority of URIs are viral in etiology [8]. For example, despite guidelines recommending against antibiotic therapy for most patients with acute uncomplicated bronchitis, studies indicate that antibiotics may be prescribed in as many as 70%–85% of outpatient bronchitis encounters [9, 10]. Even for viral URIs with a known etiology, antibiotic prescribing remains an issue, with 1 study noting a prescribing rate of 29% in patients positive for influenza without findings of pneumonia [11].

Numerous antimicrobial stewardship interventions have been effective in reducing unnecessary URI-related antibiotic prescribing [12]. Examples of interventions include peer comparison reports, provider education, patient education, order preference list modifications, antibiotic commitment posters, viral prescription pads, and electronic order sets [13–16]. Although the literature supports outpatient antibiotic stewardship practices, few studies evaluate (1) the durability of intervention(s), (2) the impact of intervention(s) across various geographic sites, (3) control outcomes (eg, rates of repeat healthcare contact for URI with and without antibiotic prescriptions), and (4) factors associated with ongoing, unnecessary/inappropriate antibiotic prescribing following initial intervention.

We aim to evaluate the frequency of unnecessary antibiotic prescribing for URI and the rate of repeat respiratory-related healthcare contact before and after a multifaceted, health system–wide antimicrobial stewardship initiative. Furthermore, we aim to determine patient, provider, and encounter-level factors associated with continued unnecessary antibiotic prescribing in the postimplementation period.

## METHODS

### Patient Consent Statement

Patient consent was not required as this retrospective cohort study was deemed exempt by the Mayo Clinic institutional review board (IRB number 23–001010) and was reported following the Strengthening the Reporting of Observational Studies in Epidemiology (STROBE) guideline for cohort studies.

### Study Design, Setting, and Interventions

This quasi-experimental, pre/post retrospective cohort study spanned 1 January 2019 to 31 December 2022. Starting 1 January 2020, Mayo Clinic implemented a comprehensive, Enterprise-wide outpatient ASP aimed at reducing unnecessary antibiotic prescribing for URIs not expected to benefit from antibiotic therapy. The Mayo Clinic Enterprise consists of 3 major destination medical centers in Rochester (Minnesota), Arizona, and Florida, along with the Mayo Clinic Health System, a network of hospitals and clinics throughout 4 regions in Minnesota and Wisconsin (southwest Wisconsin, northwest Wisconsin, southeast Minnesota, and southwest Minnesota). The initiative targeted 3 ambulatory care specialties, including primary care (ie, family medicine, community-internal medicine, women's health internal medicine, and community pediatrics and adolescent medicine), urgent care, and emergency medicine.

A multifaceted bundle of programmatic ASP interventions was implemented across the Enterprise in a stepwise fashion beginning 1 July 2020. Specific interventions included standardized provider education, dissemination of patient handouts promoting symptomatic management (ie, Viral Rx pad [17]), development of a syndrome-based, prepopulated ambulatory order panel (ie, clinical decision support tool, see Supplementary Materials), a patient-facing antibiotic commitment poster, peer comparison reporting, and a provider-facing data dashboard to facilitate self-auditing of cases where prescribing was flagged as unnecessary [17, 18]. Regional efforts were administered by local ASPs, consisting of at least 1 pharmacist and/or 1 physician trained in infectious diseases and antimicrobial stewardship.

### Participants, Data Collection, and Outcomes

Respiratory *International Classification of Diseases, 10th Revision* (*ICD-10*) diagnosis codes were grouped into tiers according to whether antibiotics are always indicated (Tier 1), sometimes indicated (Tier 2), or never indicated (Tier 3) as previously described (see Supplementary Materials) [8, 17]. Given lack of standardization across the full dataset and concerns for skewing of the data, coronavirus disease 2019 (COVID-19)–related diagnosis codes were excluded. All ambulatory encounters, including both in-person and virtual, with Tier 3 primary diagnosis codes from primary care, emergency medicine, and urgent care departments were eligible for inclusion. Minnesota patients required documentation of the Minnesota Research Authorization. Encounters with secondary diagnoses that included Tier 1 or Tier 2 URI codes were excluded. For patients with multiple encounters, each unique encounter was included if inclusion criteria were met.

Data were extracted from an electronic health record database and an institutional data warehouse. Data elements included encounter (eg, region, encounter type, encounter date, and *ICD-10* billing code), patient (eg, age, sex, race/ethnicity, antibiotic prescription, Charlson comorbidity index [CCI] [19], respiratory comorbidities), and provider features (eg, provider type, department), as well as encounter season. Patient demographic data were self-identified and included to ascertain the influence these characteristics on prescribing patterns and overall healthcare utilization. Monthly encounter volumes were determined in the postimplementation cohort to assess the impact of overall encounter volumes (ie, clinic workload) on unnecessary antibiotic prescribing. Monthly encounter volumes for all clinic encounters (including nonrespiratory encounters) were averaged for each specific clinic during the postimplementation period to determine a baseline or “expected” number of total monthly encounters. Monthly clinic encounter volumes were then compared to this baseline and converted to ordinal values of low (>1 standard deviation [SD] below mean), mild (between 0 and 1 SD below mean), moderate (between 0 and 1 SD above mean), and high (>1 SD above mean) volume for each month. Thus, high clinic volume months indicate busier months, whereas low clinic volume months indicate less busy months.

The primary outcome was the percentage of Tier 3 encounters that resulted in an antibiotic prescription. Secondary outcomes included the rate of repeat respiratory-related healthcare contact (ie, hospitalization, clinic visits, and emergency encounters) within 14 days of the index visit and patient, provider, and encounter-level factors associated with unnecessary prescribing or repeat respiratory-related healthcare contact after implementation of the intervention bundle.

### Statistical Analysis

Study periods were defined as preimplementation (1 January 2019–30 June 2020), implementation/washout (1 July 2020–30 June 2021), and postimplementation (1 July 2021–31 December 2022).

Means and standard errors are presented for continuous variables and frequencies, and percentages are presented for categorical variables. Pearson χ^2^ test was used to compare pre- and postintervention periods for antibiotic prescription and 14-day repeat respiratory-related healthcare contact. These comparisons were done for the overall sample and within a priori–specified patient, provider, and encounter-level subgroups.

Univariate and multivariable logistic regression models were fit in the postimplementation cohort to identify patient, provider, and encounter-level factors associated with both antibiotic prescribing and with 14-day repeat healthcare contact for respiratory indications. Gradient boosting machine models were used to estimate the relative influence of individual variables to identify smaller models that retained most of the predictive ability. A complete-case analysis was done in the case of missing data. R statistical software version 4.1 was utilized for all analysis (R Foundation for Statistical Computing, Vienna, Austria). Statistical significance was defined as *P* < .05.

## RESULTS

**Figure 1.**
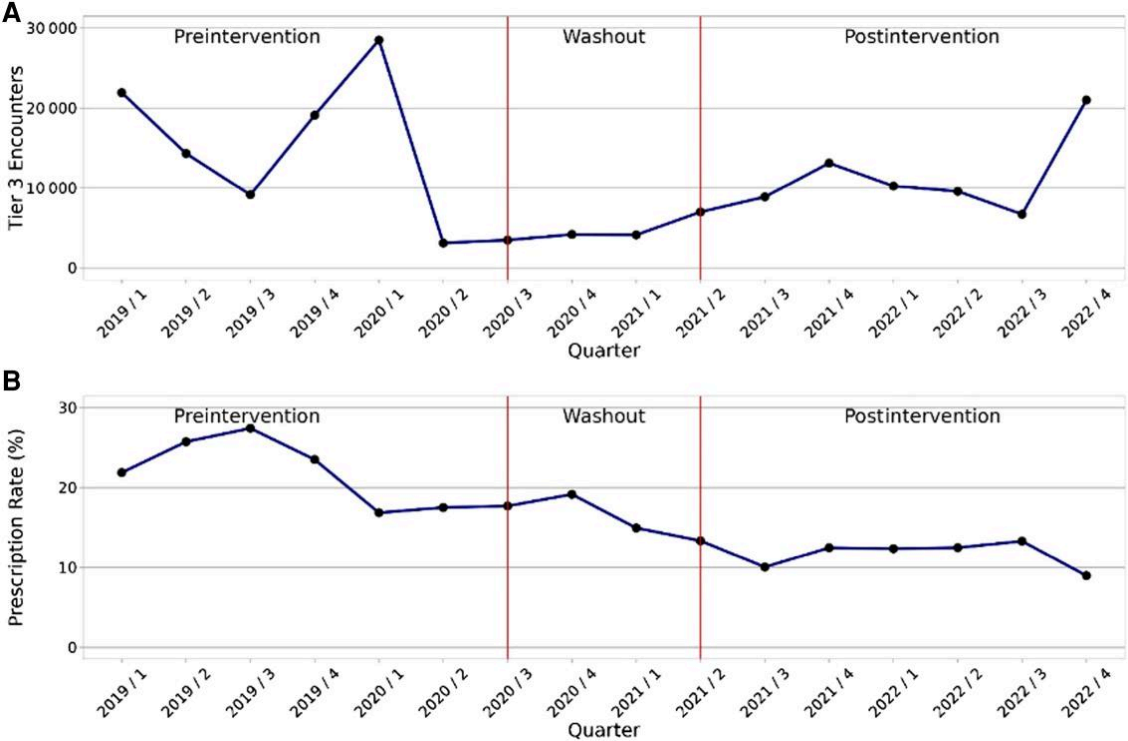
*A*, Total number of Tier 3 (never prescribe) ambulatory encounters over time by quarter across intervention periods. *B*, Tier 3 encounter antibiotic prescribing rate over time by quarter across intervention periods.

There was a total of 511 196 respiratory encounters across the Mayo Clinic Enterprise between 1 January 2019 and 31 December 2022. Of these, 184 417 Tier 3 encounters were eligible for inclusion, including 96 125, 18 759, and 69 533 in the preintervention, implementation/washout, and postintervention periods, respectively (Figure 1).

Baseline characteristics were similar between pre- and postintervention periods (Table 1). Approximately 45% of encounters were patients aged ≤18 years, with 20% of overall encounters among patients aged between 0 and 2 years. Most patients (89.4%) identified as White, while just over half were female (56.1%). Overall, 15.6% of all encounters included patients with at least 1 significant preexisting respiratory condition (ie, asthma, cystic fibrosis, pulmonary fibrosis, and bronchiectasis). Most encounters (68.8%) occurred between October and March. Primary care settings accounted for most visits (45.8%), while urgent care and emergency medicine accounted for 29.4% and 24.8% of overall encounters, respectively. Telehealth visits were more frequent in the post- compared to the preintervention period at 8.1% versus 3.5%, respectively.

**Table 1.** Baseline Characteristics Among Encounters in the Pre- and Postintervention Periods

Characteristic	Preintervention (n = 96 125)	Postintervention (n = 69 533)	Total (n = 165 658)
Age, mean (std error)	30.52 (0.08)	26.99 (0.10)	29.04 (0.06)
Age group, y			
0–2	17 824 (18.5)	15 368 (22.1)	33 192 (20.0)
3–18	22 674 (23.6)	18 764 (27.0)	41 438 (25.0)
19–65	43 876 (45.6)	27 743 (39.9)	71 619 (43.2)
>65	11 751 (12.2)	7658 (11.0)	19 409 (11.7)
Sex^a^			
Female	54 226 (56.4)	38 771 (55.8)	92 997 (56.1)
Male	41 886 (43.6)	30 756 (44.2)	72 642 (43.9)
Nonbinary	5 (0.0)	4 (0.0)	9 (0.0)
Race^b^			
American Indian/Alaska Native	416 (0.4)	376 (0.5)	792 (0.5)
Asian	2496 (2.6)	1922 (2.8)	4418 (2.7)
Black or African American	4391 (4.6)	3696 (5.4)	8087 (5.0)
Native Hawaiian/Pacific Islander	188 (0.2)	186 (0.3)	374 (0.2)
Other	2329 (2.5)	1265 (1.8)	3594 (2.2)
White	84 847 (89.6)	60 979 (89.1)	145 826 (89.4)
Encounter Season			
Apr–Sep	26 576 (27.6)	25 160 (36.2)	51 736 (31.2)
Oct–Mar	69 549 (72.4)	44 373 (63.8)	113 922 (68.8)
Pulmonary comorbidities			
Any	17 678 (18.4)	8216 (11.8)	25 894 (15.6)
Asthma	15 971 (16.6)	6999 (10.1)	22 970 (13.9)
Cystic fibrosis	40 (0.0)	31 (0.0)	71 (0.0)
Pulmonary fibrosis	2236 (2.3)	1273 (1.8)	3509 (2.1)
Bronchiectasis	678 (0.7)	484 (0.7)	1162 (0.7)
Provider type			
APP	51 453 (53.5)	38 570 (55.5)	90 023 (54.3)
Physician	44 003 (45.8)	28 813 (41.4)	72 816 (44.0)
Trainee	669 (0.7)	2150 (3.1)	2819 (1.7)
Region			
Arizona	4622 (4.8)	2843 (4.1)	7465 (4.5)
Florida	5817 (6.1)	3842 (5.5)	9659 (5.8)
MCHS NW WI	17 652 (18.4)	12 143 (17.5)	29 795 (18.0)
MCHS SE MN	21 539 (22.4)	16 332 (23.5)	37 871 (22.9)
MCHS SW MN	15 981 (16.6)	12 019 (17.3)	28 000 (16.9)
MCHS SW WI	10 246 (10.7)	7283 (10.5)	17 529 (10.6)
Rochester, MN	20 268 (21.1)	15 071 (21.7)	35 339 (21.3)
Department specialty			
Primary care	44 395 (46.2)	31 472 (45.3)	75 867 (45.8)
Urgent care	31 372 (32.6)	17 370 (25.0)	48 742 (29.4)
Emergency medicine	20 358 (21.2)	20 691 (29.8)	41 049 (24.8)
Telehealth visit	3401 (3.5)	5655 (8.1)	9056 (5.5)
Primary diagnosis			
Bronchitis/bronchiolitis	15 429 (16.1)	8354 (12.0)	23 783 (14.4)
Influenza	10 950 (11.4)	6048 (8.7)	16 998 (10.3)
Laryngitis/pharyngitis	2716 (2.8)	2747 (4.0)	5463 (3.3)
Other	61 (0.1)	69 (0.1)	130 (0.1)
Rhinitis	6527 (6.8)	5503 (7.9)	12 030 (7.3)
Serous AOM/ear disorders	8349 (8.7)	6122 (8.8)	14 471 (8.7)
URI unspecified	52 093 (54.2)	40 690 (58.5)	92 783 (56.0)
Total monthly clinic encounter volume			
Low	…	8338 (12.0)	8338 (12.0)
Mild	…	19 779 (28.4)	19 779 (28.4)
Moderate	…	24 489 (35.2)	24 489 (35.2)
High	…	16 927 (24.3)	16 927 (24.3)
Afternoon encounter^c^	52 606 (55.8)	39 174 (57.2)	91 780 (56.4)
CCI score, mean (std error)	1.22 (0.01)	1.06 (0.01)	1.15 (0.01)
0	51 071 (53.1)	41 456 (59.6)	92 527 (55.9)
1–2	30 936 (32.2)	19 145 (27.5)	50 081 (30.2)
3–4	6880 (7.2)	4301 (6.2)	11 181 (6.7)
≥5	7238 (7.5)	4631 (6.7)	11 869 (7.2)

Results are shown as No. (%) unless otherwise specified.

Abbreviations: APP, advanced practice provider; CCI, Charlson comorbidity index; MCHS, Mayo Clinic Health System; MN, Minnesota; NW, northwest; AOM, acute otitis media; SE, southeast; SW, southwest; URI, upper respiratory infection; WI, Wisconsin.

^a^10 missing values (8 pre and 2 post).

^b^2567 missing values (1458 pre and 1109 post).

^c^3000 missing values (1903 pre and 1097 post).

### Antibiotic Prescribing

Antibiotic prescribing for Tier 3 indications decreased from 21.7% in the preintervention period to 11.2% in the postintervention period (*P* < .001; Table 2, Figure 1). Significant reductions in prescribing were observed among all geographic regions and departments (*P* < .001 for all comparisons), with the largest improvement noted among urgent care clinics (51.8% relative reduction, from 24.5% to 11.8%). Rates of specific antibiotics prescribed in each period are supplied in Supplementary Table 1. Overall, the relative frequency of specific antibiotic prescriptions shifted following the intervention, with azithromycin accounting for a lower percentage of all antibiotic prescriptions in the postimplementation period (47.1% preimplementation vs 30.4% postimplementation), whereas β-lactam prescription frequency increased.

**Table 2.** Antibiotic Prescribing Among Tier 3 Respiratory Encounters in the Pre- and Postintervention Periods, Overall and by Subgroup

Antibiotic Prescribing	Preintervention (n = 96 125)	Postintervention (n = 69 533)	*P* Value
Overall	20 846 (21.7)	7776 (11.2)	<.001
Region			
Arizona	1241/4622 (26.8)	525/2843 (18.5)	<.001
Florida	2199/5817 (37.8)	477/3842 (12.4)	<.001
MCHS NW WI	4073/17 652 (23.1)	1750/12 143 (14.4)	<.001
MCHS SE MN	6023/21 539 (28.0)	2544/16 332 (15.6)	<.001
MCHS SW MN	3841/15 981 (24.0)	848/12 019 (7.1)	<.001
MCHS SW WI	2012/10 246 (19.6)	716/7283 (9.8)	<.001
Rochester, MN	1457/20 269 (7.2)	916/15 071 (6.1)	<.001
Department specialty			
Primary care	10 119/44 395 (22.8)	3917/31 472 (12.4)	<.001
Urgent care	7673/31 372 (24.5)	2049/17 370 (11.8)	<.001
Emergency medicine	3054/20 358 (15.0)	1810/20 691 (8.7)	<.001
Encounter type			
In-person	20 579/92 724 (22.2)	7591/63 878 (11.4)	<.001
Telehealth	267/3401 (7.9)	485/5655 (8.6)	.225
Provider type			
APP	11 436/51 453 (22.2	4374/38 570 (11.3)	<.001
Physician	9316/44 003 (21.2)	3144/28 813 (10.9)	<.001
Trainee	94/669 (14.1)	258/2150 (12.0)	.161
Age group, y			
0–2	2414/17 824 (13.5)	1368/15 368 (8.9)	<.001
3–18	3736/22 674 (16.5)	1699/18 764 (9.1)	<.001
19–65	10 645/43 876 (24.3)	3353/27 743 (12.1)	<.001
>65	4051/11 751 (34.5)	1356/7658 (17.7)	<.001
Primary diagnosis			
Bronchitis/bronchiolitis	8509/15 429 (55.1)	2350/8354 (28.1)	<.001
Influenza	502/10 950 (4.6)	148/6048 (2.4)	<.001
Laryngitis/pharyngitis	122/2716 (4.5)	89/2747 (3.2)	.016
Other	11/61 (18.0)	11/69 (15.9)	.751
Rhinitis	366/6527 (5.6)	183/5503 (3.3)	<.001
Serous AOM/ear disorders	4548/8349 (54.5)	2578/6122 (42.1)	<.001
URI unspecified	6788/52 093 (13.0)	2417/40 690 (5.9)	<.001
CCI score			
0	7668/51 071 (15.0)	3638/41 456 (8.8)	<.001
1–2	8499/30 936 (27.5)	2549/19 145 (13.3)	<.001
3–4	2209/6880 (32.1)	737/4301 (17.1)	<.001
≥5	2470/7238 (34.1)	852/4631 (18.4)	<.001

Results are shown as No. (%) unless otherwise specified.

Abbreviations: APP, advanced practice provider; CCI, Charlson comorbidity index; MCHS, Mayo Clinic Health System; MN, Minnesota; NW, northwest; AOM, acute otitis media; SE, southeast; SW, southwest; URI, upper respiratory infection; WI, Wisconsin.

### Repeat Respiratory-Related Healthcare Contact

Repeat healthcare contact for respiratory conditions within 14 days of index encounter was less common when an antibiotic was prescribed in the overall cohort (6.9% antibiotics vs 9.7% no antibiotic, *P* < .001; Supplementary Table 2). This finding was consistent in both the pre- and postintervention cohorts. The rate of repeat respiratory-related healthcare contact in those receiving antibiotic prescriptions was numerically higher but not significantly different in the postintervention compared to the preintervention cohort (6.7% pre vs 7.3% post, *P* = .116). Repeat respiratory-related healthcare contact was less common in the postimplementation cohort among encounters where an antibiotic was not prescribed (9.9% preintervention vs 9.4% postintervention, *P* = .004).

### Predictors of Antibiotic Prescribing in the Postimplementation Cohort

In the univariate model (Supplementary Table 3), increasing patient age and CCI were strongly associated with increased likelihood of antibiotic prescribing (age >65: odds ratio [OR], 2.20 [95% confidence interval {CI}, 2.03–2.39]; CCI ≥5: OR, 2.34 [95% CI, 2.16–2.54]; *P* < .001 for both comparisons). All concomitant respiratory conditions, excluding cystic fibrosis, were associated with antibiotic prescribing. Interestingly, higher total monthly clinic encounter volumes were associated with lower antibiotic prescribing (OR, 0.67 [95% CI, .62–.73]; *P* < .001). Relative to bronchitis/bronchiolitis, serous acute otitis media (AOM)/ear disorders had a higher rate of antibiotic prescribing (OR, 1.86 [95% CI, 1.73–1.99]; *P* < .001), whereas influenza, laryngitis/pharyngitis, rhinitis, and unspecified URI were associated with lower prescribing rates.

In the multivariable model, advancing age, increasing CCI, and serous acute otitis media (AOM)/ear disorders were associated with antibiotic prescribing, whereas high total monthly clinic encounter volume was associated with less antibiotic prescribing (Table 3). No difference was noted among provider type. Afternoon encounters were marginally associated with antibiotic prescribing (OR, 1.06 [95% CI, 1.01–1.12]; *P* = .027). Males were more likely to receive an antibiotic prescription compared to females in the overall model (OR, 1.08 [95% CI, 1.02–1.14]; *P* = .007). Patients identifying as Asian, Black or African American, or other race were slightly less likely to receive antibiotic prescriptions relative to White patients. The top 3 variables influencing antibiotic prescribing included the primary diagnosis (ie, syndrome) from index encounter, patient age, and CCI, which accounted for >95% of relative influence (Supplementary Figure 1), suggesting that persistent prescribing can be largely predicted by these 3 factors. This is also supported by the closeness of the area under the receiver operating characteristic (ROC) curves from the 2-multivariable models (0.799 for the full model vs 0.795 for the 3-variable model).

**Table 3.** Multivariable Models for Antibiotic Prescriptions in the Postintervention Cohort

Characteristic	All Variables (n = 67 341)			Top 3 (n = 69 533)		
	OR	(95% CI)	*P* Value	OR	(95% CI)	*P* Value
Age group, y						
0–2	…	…		…	…	
3–18	1.28	(1.18–1.40)	<.001	1.25	(1.15–1.36)	<.001
19–65	1.51	(1.39–1.64)	<.001	1.43	(1.32–1.56)	<.001
>65	1.90	(1.70–2.13)	<.001	1.93	(1.73–2.15)	<.001
Male sex	1.08	(1.02–1.14)	.007	…	…	
Race						
White	…	…		…	…	
American Indian/Alaska Native	0.75	(.50–1.10)	.2	…	…	
Asian	0.81	(.67–.97)	.027	…	…	
Black or African American	0.84	(.73–.96)	.012	…	…	
Native Hawaiian/Pacific Islander	0.87	(.48–1.47)	.6	…	…	
Other	0.74	(.58–.94)	.015	…	…	
Encounter season						
Apr–Sep	…	…		…	…	
Oct–Mar	1.06	(1.00–1.12)	.033	…	…	
Asthma	0.95	(.87–1.04)	.3	…	…	
Cystic fibrosis	1.95	(.54–5.38)	.2	…	…	
Pulmonary fibrosis	1.08	(.91–1.28)	.4	…	…	
Bronchiectasis	1.57	(1.22–2.01)	<.001	…	…	
Provider type						
APP	…	…		…	…	
Physician	1.06	(1.0–1.12)	.074	…	…	
Trainee	1.12	(.96–1.29)	.15	…	…	
Department specialty						
Primary care	…	…		…	…	
Urgent care	0.86	(.80–.92)	<.001	…	…	
Emergency medicine	0.95	(.88–1.02)	.13	…	…	
Telehealth visit	1.07	(.96–1.19)	.2	…	…	
Primary diagnosis						
Bronchitis/bronchiolitis	…	…		…	…	
Influenza	0.07	(.06–.08)	<.001	0.07	(.06–.08)	<.001
Laryngitis/pharyngitis	0.11	(.09–.14)	<.001	0.11	(.09–.14)	<.001
Other	0.47	(.23–.87)	.025	0.47	(.23–.88)	.026
Rhinitis	0.08	(.07–.09)	<.001	0.08	(.07–.09)	<.001
Serous AOM/ear disorders	2.27	(2.10–2.46)	<.001	2.23	(2.07–2.40)	<.001
URI unspecified	0.18	(.17–.20)	<.001	0.18	(.17–.19)	<.001
Total monthly clinic encounter volume						
Low	…	…		…	…	
Mild	0.95	(.88–1.04)	.3	…	…	
Moderate	0.93	(.85–1.01)	.082	…	…	
High	0.76	(.69–.83)	<.001	…	…	
Encounter time						
Morning	…	…		…	…	
Afternoon	1.06	(1.01–1.12)	.027	…	…	
CCI score						
0	…	…		…	…	
1–2	1.38	(1.29–1.49)	<.001	1.39	(1.30–1.48)	<.001
3–4	1.71	(1.53–1.91)	<.001	1.75	(1.57–1.94)	<.001
≥5	1.72	(1.53–1.93)	<.001	1.82	(1.63–2.02)	<.001

Abbreviations: APP, advanced practice provider; CCI, Charlson comorbidity index; CI, confidence interval; AOM, acute otitis media; OR, odds ratio; URI, respiratory infection.

### Predictors of 14-Day Repeat Respiratory-Related Healthcare Contact in the Postimplementation Cohort

In the univariate model for 14-day repeat respiratory-related healthcare contact, relative to encounters of patients aged 0–2 years, all other age groups were less likely to return for a respiratory indication within 14 days (Supplementary Table 4). Additionally, urgent care, emergency medicine, telehealth, high monthly total clinic encounter volumes, patients with concomitant asthma, and CCI scores ≥5 were more likely to result in repeat healthcare contact. Receipt of an antibiotic prescription was associated with a lower rate of repeat contact (OR, 0.74 [95% CI, .67–.81]; *P* < .001), but was not one of the most important variables according to relative influence (Supplementary Figure 2). No differences were noted among provider type, patient sex, or patient race.

Three different multivariable models were fit for repeat respiratory-related healthcare contact comprising all, top 8, and top 5 variables by relative influence (Table 4, Supplementary Figure 2). When limiting the model to only the top 5 contributing variables, telehealth visit, department specialty, primary diagnosis, CCI, and age group had the most influence on repeat respiratory-related 14-day healthcare contact. When comparing the multivariable models on area under the ROC curve, they are similar (full model = 0.644, 8-variable model = 0.640, 5-variable model = 0.636), indicating that a reduced set of predictors is sufficient.

**Table 4.** Multivariable Models for 14-Day Repeat Respiratory-Related Healthcare Contact in the Postintervention Cohort

Characteristic	All Variables (n = 67 341)			Top 8 (n = 68 436)			Top 5 (n = 69 533)		
	OR	(95% CI)	*P* Value	OR	(95% CI)	*P* Value	OR	(95% CI)	*P* Value
Age group, y									
0–2	…	…		…	…		…	…	
3–18	0.48	(.45–.52)	<.001	0.48	(.45–.52)	<.001	0.49	(.46–.53)	<.001
19–65	0.35	(.32–.37)	<.001	0.35	(.32–.38)	<.001	0.35	(.32–.38)	<.001
>65	0.36	(.32–.41)	<.001	0.37	(.32–.41)	<.001	0.36	(.32–.40)	<.001
Male sex	0.93	(.88–.98)	.007	…	…		…	…	
Race									
White	…	…		…	…		…	…	
American Indian/Alaska Native	1.05	(.73–1.46)	.8	…	…		…	…	
Asian	0.90	(.76–1.06)	.2	…	…		…	…	
Black or African American	0.87	(.77–.98)	.023	…	…		…	…	
Native Hawaiian/Pacific Islander	0.72	(.39–1.20)	.2	…	…		…	…	
Other	0.87	(.70–1.06)	.2	…	…		…	…	
Encounter season									
Apr–Sep	…	…		…	…		…	…	
Oct–Mar	1.04	(.98–1.10)	.2	…	…		…	…	
Asthma	1.26	(1.15–1.39)	<.001	1.27	(1.16–1.40)	<.001	…	…	
Cystic fibrosis	0.55	(.09–1.86)	.4	…	…		…	…	
Pulmonary fibrosis	1.24	(1.02–1.51)	.029	…	…		…	…	
Bronchiectasis	0.99	(.72–1.34)	>.9	…	…		…	…	
Provider type									
APP	…	…		…	…		…	…	
Physician	0.92	(.86–.97)	.004	…	…		…	…	
Trainee	1.23	(1.05–1.43)	.010	…	…		…	…	
Department specialty									
Primary care	…	…		…	…		…	…	
Urgent care	1.25	(1.16–1.34)	<.001	1.28	(1.20–1.38)	<.001	1.34	(1.25–1.44)	<.001
Emergency medicine	1.56	(1.45–1.67)	<.001	1.52	(1.42–1.62)	<.001	1.57	(1.47–1.68)	<.001
Telehealth visit	1.77	(1.59–1.97)	<.001	1.77	(1.59–1.96)	<.001	1.93	(1.76–2.12)	<.001
Primary diagnosis									
Bronchitis/bronchiolitis	…	…		…	…		…	…	
Influenza	0.78	(.69–.88)	<.001	0.83	(.74–.93)	.002	0.85	(.75–.95)	.004
Laryngitis/pharyngitis	0.79	(.69–.91)	.001	0.83	(.72–.96)	.011	0.83	(.72–.95)	.008
Other	1.11	(.51–2.15)	.8	1.11	(.51–2.15)	.8	1.12	(.51–2.17)	.8
Rhinitis	0.32	(.27–.38)	<.001	0.33	(.28–.40)	<.001	0.38	(.32–.44)	<.001
Serous AOM/ear disorders	0.80	(.71–.91)	<.001	0.77	(.68–.87)	<.001	0.77	(.68–.87)	<.001
URI unspecified	0.80	(.74–.87)	<.001	0.86	(.79–.93)	<.001	0.86	(.80–.93)	<.001
Total monthly clinic encounter volume									
Low	…	…		…	…		…	…	
Mild	0.96	(.87–1.06)	.4	0.96	(.87–1.05)	.4	…	…	
Moderate	1.10	(1.01–1.21)	.039	1.10	(1.01–1.21)	.040	…	…	
High	1.18	(1.08–1.30)	<.001	1.19	(1.09–1.31)	<.001	…	…	
Encounter time									
Morning	…	…		…	…		…	…	
Afternoon	1.01	(.96–1.06)	.8	1.01	(.95–1.06)	.8	…	…	
CCI score									
0	…	…		…	…		…	…	
1–2	1.32	(1.23–1.43)	<.001	1.31	(1.22–1.42)	<.001	1.40	(1.31–1.50)	<.001
3–4	1.68	(1.47–1.91)	<.001	1.67	(1.47–1.90)	<.001	1.75	(1.54–1.98)	<.001
≥5	1.97	(1.73–2.25)	<.001	1.99	(1.75–2.26)	<.001	2.08	(1.84–2.35)	<.001
Antibiotic prescribed	0.74	(.67–.81)	<.001	…	…		…	…	

Abbreviations: APP, advanced practice provider; CCI, Charlson Comorbidity Index; CI, confidence interval; AOM, acute otitis media; OR, odds ratio; URI, respiratory infection.

## DISCUSSION

Our study adds to mounting evidence that targeted outpatient antibiotic stewardship programs are effective at reducing unnecessary or inappropriate antibiotic prescribing for URIs [12, 13, 15, 16, 20–24]. We observed a 48.4% relative reduction in prescriptions for Tier 3 URI following comprehensive outpatient antimicrobial stewardship program implementation, amounting to approximately 7300 unnecessary antibiotic prescriptions avoided. This finding was consistent among all specialties, regions, providers, and patient groups, thereby increasing external validity. Encounters where an antibiotic was prescribed were less likely to result in repeat respiratory-related healthcare contact for a respiratory indication within 14 days of index visit in the overall, pre-, and postintervention cohorts; however, the rate of repeat contact in patients who did not receive antibiotic therapy slightly decreased following implementation of the multifaceted intervention.

The decision of whether to prescribe or withhold an antibiotic is complicated, encompassing not only clinical, but also mutually dependent nonclinical factors, such as patient, provider, and healthcare system–related factors [25]. Even time of day has been correlated with the decision to prescribe antibiotics, with later appointments more likely to result in antibiotic prescription than earlier appointments, a phenomenon likely attributable to decision fatigue [26]. Socioeconomic patient factors not assessed in this report, including access to clinics and/or clinic location, may additionally impact prescribing decisions. Given the complexity of these influential factors, determining the relative magnitude of each is important to provide clarity to outpatient ASPs, particularly in the context of persistent inappropriate prescribing following intervention.

Using univariate and multivariable regression models in the postimplementation cohort, we were able to identify multiple factors impacting the decision to prescribe antibiotics. Consistent with prior research, we too found that afternoon appointments were more likely to result in unnecessary antibiotic prescriptions, even when controlling for other variables in the multivariate model. Interestingly, busier clinic months were associated with lower rates of unnecessary prescribing. One explanation for this might be the seasonality of cold and flu season, when encounter volumes peak and providers simultaneously feel more confident in attributing symptoms to circulating viruses. However, while encounter-level factors were influential in the overall model, we found that the most significant factors impacting the decision to prescribe antibiotics by relative influence are specific diagnosis, patient age, and patient comorbidities. Serous AOM/ear disorders were the diagnoses most strongly associated with antibiotic prescribing (OR, 2.27 [95% CI, 2.10–2.46]), which could indicate diagnostic uncertainty, a knowledge deficit, or diagnostic coding issues, all of which can be addressed with targeted follow-up interventions. The influence of advancing age and higher CCI on the propensity to prescribe antibiotics is not surprising in the context of the well-known influence of fear in prescribing decisions. In this patient population, fear of complications secondary to “missing” a bacterial diagnosis may be clouding the complete risk/benefit picture, wherein antibiotic-related complications, microbiota disruptions, and the development of antibiotic resistance are viewed as less common, less severe, and/or more abstract [27, 28].

Of particular importance is the association between antibiotic prescriptions and repeat respiratory-related 14-day respiratory healthcare contact, which was consistent in the overall, pre-, and postintervention cohorts. Previous studies have documented that antibiotic prescriptions are associated with higher patient satisfaction scores [29, 30], particularly if patients enter encounters expecting an antibiotic prescription [31]. It is possible that lack of antibiotic prescription drove certain patients (ie, the inconvincible patients) [32] to seek prescriptions elsewhere within the health system, thereby driving repeat contact. Such behaviors might be mitigated through patient education on diagnosis (eg, “you have a viral illness for which antibiotics have no proven benefit but may cause harm”), expectations (eg, “you may experience a lingering cough for up to 3 weeks”), and symptomatic management (eg, “take pseudoephedrine 60 mg by mouth every 4–6 hours as needed for congestion”). We provided a tool to providers (ie, Viral Rx pad) with patient education and preselected recommendations for over-the-counter medications to manage specific symptoms. This was available in multiple languages, and accessible in both in print and prepopulated progress note formats, with links directly to the resource from the prepopulated ambulatory order panel/clinical decision support tool.

The COVID-19 pandemic impacted the healthcare system in dramatic ways, including reduced clinic encounter volumes, hyperawareness of viral causes of URI, and a shift to telemedicine [33, 34]. In our study, we chose to exclude all encounters with COVID-19–related diagnosis codes. Even though antibiotics are not indicated for COVID-19, including these encounters in the overall denominators resulted in a very dramatic and clearly artificial drop in the prescribing rate, which coincided with fluctuations of COVID-19 encounters [35]. We also found increased use of telemedicine in the postimplementation (post–July 2021) cohort. Importantly, telehealth visits were associated with an increased rate of repeat respiratory-related healthcare contact, representing the fourth most important factor by relative influence behind patient age, department specialty, and primary diagnosis; however, we are unable to fully elucidate whether these repeat healthcare contact events represent planned in-person follow-ups or unplanned encounters for ongoing respiratory syndromes.

### Limitations

Our study has several limitations, the most prevalent being a retrospective design and lack of randomization, which limits the ability to attribute causality of the intervention. Importantly, this study and the intervention period coincided with the COVID-19 pandemic, which could have influenced the study results in unmeasurable ways; however, as mentioned previously, COVID-19–related diagnoses were excluded from the analysis to limit the impact of an inflated denominator on Tier 3 prescribing rates. Utilizing encounter *ICD-10* diagnosis codes may have resulted in some misclassification bias, including with use of unspecified codes, which comprised a large portion of our cohort (56% overall). However, diagnosis codes represent the most utilized method to classify encounters in this type of retrospective work, and methods similar to ours have been used in prior studies [1, 8, 23]. We additionally were unable to track delayed prescriptions (ie, prescriptions intended to only be filled if symptoms failed to improve with time), which may explain why we observed a higher rate of prescribing with serous AOM. Furthermore, we only tracked prescriptions written, rather than filled, which limits our ability to account for patient adherence as a confounding factor. Our primary balancing measure of 14-day repeat respiratory-related healthcare contact was broad and lacked the specificity to determine if an antibiotic prescription might have made a difference, nor did it account for planned follow-up compared to unplanned recontact with the care system. Last, we were unable to detect the utilization of certain interventions, such as patient-level counseling strategies, which might be important in preventing repeat respiratory-related healthcare contact for persistent symptoms that are within the expected course of illness for viral infections.

## CONCLUSIONS

Implementation of a multifaceted outpatient antimicrobial stewardship initiative successfully reduced unnecessary antibiotic prescribing for URIs without increasing the rate of 14-day repeat respiratory-related healthcare contact. Persistent antibiotic prescribing after bundle implementation was noted among encounters for patients with advanced age and increasing comorbidities. Additional awareness about the risks and benefits of antibiotics in older, medically complex individuals may be helpful in further reducing unnecessary antibiotic prescriptions.

## Supplementary Material

ofad585_Supplementary_Data

## Supplementary Data

Supplementary materials are available at *Open Forum Infectious Diseases* online. Consisting of data provided by the authors to benefit the reader, the posted materials are not copyedited and are the sole responsibility of the authors, so questions or comments should be addressed to the corresponding author.

## Notes

***Acknowledgments*.** We acknowledge the contributions of Darrin Christopherson, MBA, Benjamin Anderson, PharmD, Laura Dinnes, PharmD, Nipunie Rajapakse, MD, MPH, Harry Powers, MD, Kevin Epps, PharmD, Kellie Arensman Hannan, PharmD, Sara Ausman, PharmD, Christina G. O’Connor, PharmD, Sarah Lessard, PharmD, Teresa Seville, MD, Jamilah Shubeilat, MD, and Angie Ton, MD, of the Mayo Clinic and affiliated health system sites, for their assistance in programmatic development and implementation across the Mayo Clinic Enterprise.

***Author contributions.*** D. I., R. W. S., and R. D. had full access to all the data in the study and take responsibility for the integrity of the data and the accuracy of the data analysis. Concept and design: D. I., R. W. S., K. J., A. V., P. V., and K. A. P. Acquisition, analysis, or interpretation of data: D. I. and R. W. S. Drafting of the manuscript: D. I., R. W. S., and R. D. Critical revision of the manuscript for important intellectual content: All authors. Statistical analysis: R. D. Administrative, technical, or material support: E. D. Supervision: A. V., P. V., K. J., and K. A. P.

***Financial support.*** This work was partially funded by a grant from the Mayo Midwest Pharmacy Research Committee.
