# Correction to: Evaluation of Multisite Programmatic Bundle to Reduce Unnecessary Antibiotic Prescribing for Respiratory Infections: A Retrospective Cohort Study

**DOI:** 10.1093/ofid/ofae145

**Published:** 2024-03-18

This is a correction to: Dan Ilges, Kelsey Jensen, Evan Draper, Ross Dierkhising, Kimberly A Prigge, Paschalis Vergidis, Abinash Virk, Ryan W Stevens, Evaluation of Multisite Programmatic Bundle to Reduce Unnecessary Antibiotic Prescribing for Respiratory Infections: A Retrospective Cohort Study, *Open Forum Infectious Diseases*, Volume 10, Issue 12, December 2023, ofad585, https://doi.org/10.1093/ofid/ofad585.

In the originally published version of this manuscript, the supplementary file ‘Supplementary Material Respiratory ICD10 Code Book.xlsx’ was erroneously omitted.

This error has been corrected.

