# Supplementary material for: Evaluation of Multisite Programmatic Bundle to Reduce Unnecessary Antibiotic Prescribing for Respiratory Infections: A Retrospective Cohort Study
Source: Open Forum Infect Dis. 2023 Nov 21;10(12):ofad585. doi: 10.1093/ofid/ofad585 (PMC10727194; doi:10.1093/ofid/ofad585)
Supplement: ofad585_Supplementary_Data [file ofad585_supplementary_data.zip › Supplemental_Material.docx]

**Evaluation of multisite programmatic bundle to reduce unnecessary antibiotic prescribing for respiratory infections: A retrospective cohort study**

**Supplementary Appendix**

**Authors:**

Dan Ilges, Pharm.D.

Department of Pharmacy Services, Mayo Clinic Arizona, Phoenix, Arizona, USA

Kelsey Jensen, Pharm.D.

Department of Pharmacy Services, Mayo Clinic Health System – Southeast MN, Austin, Minnesota, USA

Evan Draper, Pharm.D.

Department of Pharmacy Services, Mayo Clinic, Rochester, Minnesota, USA

Ross Dierkhising, M.S.

Division of Clinical Trials and Biostatistics, Mayo Clinic, Rochester, Minnesota, USA

Kimberly A. Prigge

Division of Family Medicine, Mayo Clinic, Rochester, Minnesota, USA

Paschalis Vergidis, M.D.

Division of Public Health, Infectious Diseases, and Occupational Medicine, Mayo Clinic, Rochester, Minnesota, USA

Abinash Virk, M.D.

Division of Public Health, Infectious Diseases, and Occupational Medicine, Mayo Clinic, Rochester, Minnesota, USA

Ryan W. Stevens, Pharm.D. (corresponding author)

Department of Pharmacy Services, Mayo Clinic, Rochester, Minnesota, USA

**Supplementary Materials Table of Contents**

**Page # – Title**

3 – Supplementary eTable 1: Antibiotic Prescriptions by Study Period Among Those with a Prescription

4 – Supplementary eTable 2: 14-Day Repeat Respiratory-Related Healthcare Contact by Antibiotic Prescription Group and Intervention Period

5 – Supplementary eTable 3: Univariate Models for Antibiotic Prescriptions in the Post-Intervention Cohort

7 – Supplementary eFigure 1: Relative Influence of Variables for Predicting Antibiotic Prescribing in the Post-Implementation Cohort

8 – Supplementary eTable 4: Univariate Models for 14-Day Repeat Healthcare Contact in the Post-Intervention Cohort

10 – Supplementary eFigure 2: Relative Influence of Variables for Predicting 14-Day Repeat Healthcare Contact in the Post-Implementation Cohort

11 – EZ ID Respiratory Order Panel (Adult)

18 – EZ ID Respiratory Order Panel (Pediatric)

**Supplementary eTable 1: Antibiotic Prescriptions by Study Period Among Those with a Prescription**

| **Antibiotic*** | Pre-Implementation (N=20846) | Post-Implementation (N=7776) | Total (N=28622) |
| --- | --- | --- | --- |
| **Azithromycin** | 9810 (47.1%) | 2362 (30.4%) | 12172 (42.5%) |
| **Amoxicillin** | 4614 (22.1%) | 2318 (29.8%) | 6932 (24.2%) |
| **Doxycycline** | 2550 (12.2%) | 1074 (13.8%) | 3624 (12.7%) |
| **Amoxicillin/clavulanate** | 2268 (10.9%) | 1253 (16.1%) | 3521 (12.3%) |
| **Cefdinir** | 1053 (5.1%) | 727 (9.3%) | 1780 (6.2%) |
| **Levofloxacin** | 455 (2.2%) | 105 (1.4%) | 560 (2.0%) |
| **Cefuroxime** | 188 (0.9%) | 29 (0.4%) | 217 (0.8%) |
| **Penicillin VK** | 39 (0.2%) | 23 (0.3%) | 62 (0.2%) |
| **Clindamycin** | 27 (0.1%) | 11 (0.1%) | 38 (0.1%) |
| **Moxifloxacin** | 6 (0.0%) | 3 (0.0%) | 9 (0.0%) |
| **Clarithromycin** | 13 (0.1%) | 1 (0.0%) | 14 (0.0%) |
| **Cefpodoxime** | 3 (0.0%) | 7 (0.1%) | 10 (0.0%) |

Results shown as no. (%)

*some encounters generated more than one prescription

**Supplementary eTable 2: 14-Day Repeat Respiratory-Related Healthcare Contact by Antibiotic Prescription Group and Intervention Period**

| **Group** | No Antibiotic Prescribed (N=137036) | Antibiotic Prescribed (N=28622) | p value |
| --- | --- | --- | --- |
| **Overall** | 13280 (9.7%) | 1966 (6.9%) | < 0.001 |
| **Pre-Intervention** | 7451/75279 (9.9%) | 1402/20846 (6.7%) | < 0.001 |
| **Post-Intervention** | 5829/61757 (9.4%) | 564/7776 (7.3%) | < 0.001 |
|  | Pre-Intervention (N=96125) | Post-Intervention (N=69533) |  |
| **Antibiotic Prescribed** | 1402/20846 (6.7%) | 564/7776 (7.3%) | 0.116 |
| **No Antibiotic Prescribed** | 7451/75279 (9.9%) | 5829/61757 (9.4%) | 0.004 |

Results shown as no. (%) unless otherwise specified

**Supplementary eTable 3: Univariate Models for Antibiotic Prescriptions in the Post-Intervention Cohort**

| **Characteristic** | OR | 95% CI | P value |
| --- | --- | --- | --- |
| **Age Group** |  |  |  |
| 0-2 | --- | --- | --- |
| 3-18 | 1.02 | 0.95, 1.10 | 0.623 |
| 19-65 | 1.41 | 1.32, 1.50 | < 0.001 |
| >65 | 2.20 | 2.03, 2.39 | < 0.001 |
| **Sex** |  |  |  |
| Female | --- | --- | --- |
| Male | 0.99 | 0.94, 1.04 | 0.652 |
| **Race** |  |  |  |
| White | --- | --- | --- |
| American Indian/Alaskan Native | 0.68 | 0.46, 0.96 | 0.039 |
| Asian | 0.61 | 0.51, 0.72 | < 0.001 |
| Black or African American | 0.60 | 0.53, 0.68 | < 0.001 |
| Native Hawaiian/Pacific Islander | 0.71 | 0.41, 1.15 | 0.191 |
| Other | 0.54 | 0.43, 0.67 | < 0.001 |
| **Encounter Season** |  |  |  |
| Apr-Sep | --- | --- | --- |
| Oct-Mar | 0.90 | 0.86, 0.95 | < 0.001 |
| **Pulmonary Comorbidity** |  |  |  |
| None | --- | --- | --- |
| Asthma | 1.18 | 1.10, 1.27 | < 0.001 |
| Cystic Fibrosis | 1.18 | 0.35, 3.01 | 0.761 |
| Pulmonary Fibrosis | 1.78 | 1.54, 2.06 | < 0.001 |
| Bronchiectasis | 2.27 | 1.82, 2.81 | < 0.001 |
| **Provider Type** |  |  |  |
| Advance Practice Provider | --- | --- | --- |
| Physician | 0.96 | 0.91, 1.01 | 0.080 |
| Trainee | 1.07 | 0.93, 1.22 | 0.349 |
| **Department Specialty** |  |  |  |
| Family Medicine | --- | --- | --- |
| Urgent Care | 0.94 | 0.89, 1.00 | 0.036 |
| Emergency Medicine | 0.67 | 0.64, 0.72 | < 0.001 |
| **Visit Type** |  |  |  |
| In-Person | --- | --- | --- |
| Telehealth | 0.73 | 0.66-0.80 | < 0.001 |
| **Primary Diagnosis** |  |  |  |
| Bronchitis/Bronchiolitis | --- | --- | --- |
| Influenza | 0.06 | 0.05, 0.08 | < 0.001 |
| Laryngitis/Pharyngitis | 0.09 | 0.07, 0.11 | < 0.001 |
| Other | 0.48 | 0.24, 0.89 | 0.028 |
| Rhinitis | 0.09 | 0.08, 0.10 | < 0.001 |
| Serous OM/Ear Disorders | 1.86 | 1.73, 1.99 | < 0.001 |
| URI unspecified | 0.16 | 0.15, 0.17 | < 0.001 |
| **Encounter Volume** |  |  |  |
| Low | --- | --- | --- |
| Mild | 0.93 | 0.86, 1.00 | 0.056 |
| Moderate | 0.89 | 0.83, 0.96 | 0.003 |
| High | 0.67 | 0.62, 0.73 | < 0.001 |
| **Encounter Time** |  |  |  |
| Morning | --- | --- | --- |
| Afternoon | 1.04 | 0.99, 1.09 | 0.115 |
| **Charlson Group** |  |  |  |
| 0 | --- | --- | --- |
| 1-2 | 1.60 | 1.51, 1.68 | < 0.001 |
| 3-4 | 2.15 | 1.97, 2.34 | < 0.001 |
| 5+ | 2.34 | 2.16, 2.54 | < 0.001 |

Results shown as no. (%) unless otherwise specified

Abbreviations: Oct, October; Mar, March; OM, otitis media; URI, upper respiratory infection.

**Supplementary eFigure 1: Relative Influence of Variables for Predicting Antibiotic Prescribing in the Post-Implementation Cohort**

**
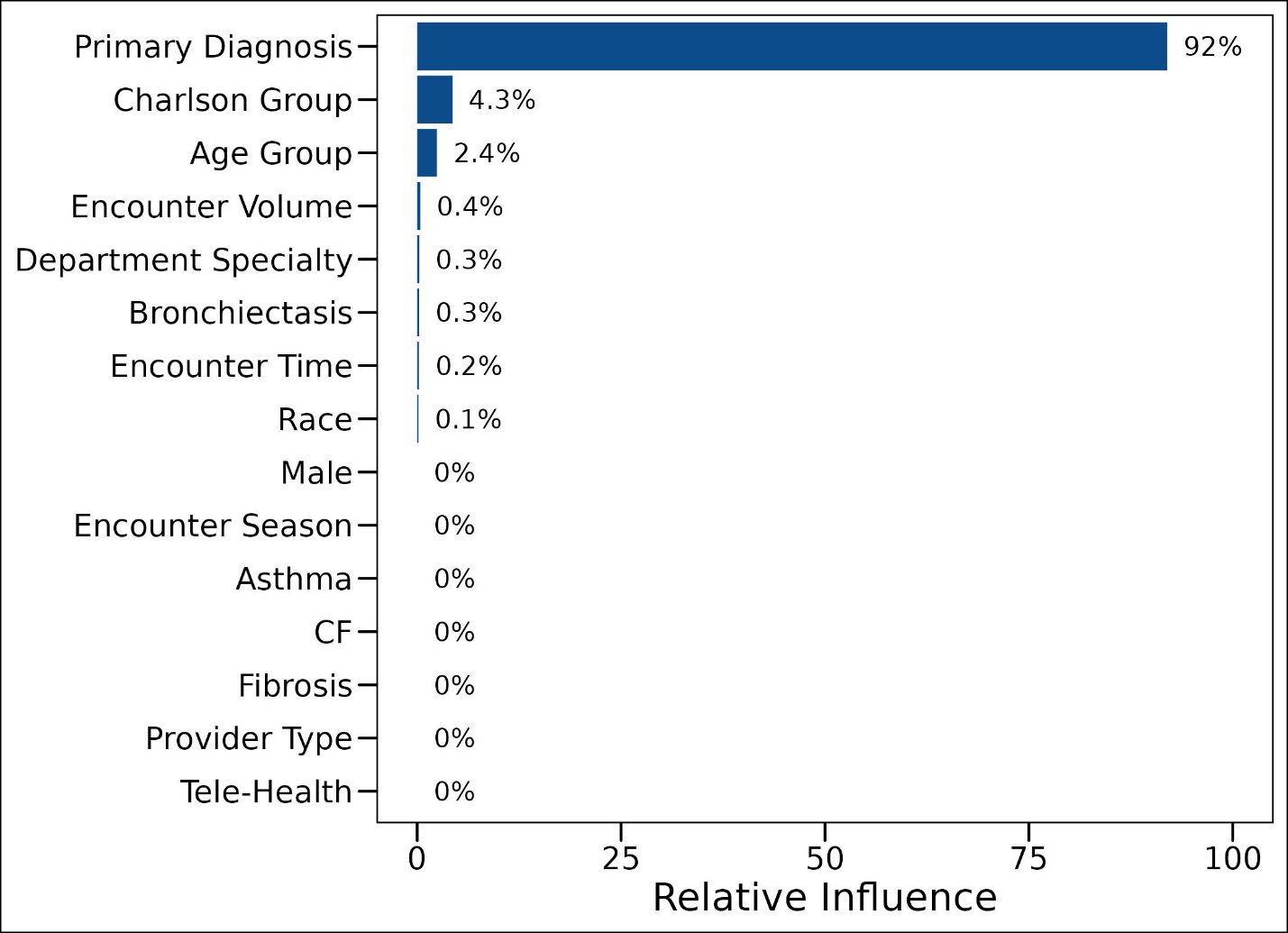
**

Abbreviations: CF, cystic fibrosis.

**Supplementary eTable 4: Univariate Models for 14-Day Repeat Respiratory-Related Healthcare Contact in the Post-Intervention Cohort**

| **Characteristic** | OR | 95% CI | P value |
| --- | --- | --- | --- |
| **Age Group** |  |  |  |
| 0-2 | --- | --- | --- |
| 3-18 | 0.51 | 0.48, 0.55 | <0.001 |
| 19-65 | 0.45 | 0.42, 0.48 | <0.001 |
| >65 | 0.53 | 0.49, 0.59 | <0.001 |
| **Sex** |  |  |  |
| Female | --- | --- | --- |
| Male | 1.02 | 0.97, 1.08 | 0.367 |
| **Race** |  |  |  |
| White | --- | --- | --- |
| American Indian/Alaskan Native | 1.07 | 0.75, 1.48 | 0.710 |
| Asian | 0.95 | 0.81, 1.11 | 0.515 |
| Black | 0.93 | 0.82, 1.04 | 0.204 |
| Native Hawaiian/Pacific Islander | 0.80 | 0.44, 1.32 | 0.411 |
| Other | 0.88 | 0.72, 1.08 | 0.234 |
| **Encounter Season** |  |  |  |
| Apr-Sep | --- | --- | --- |
| Oct-Mar | 1.09 | 1.03, 1.15 | 0.002 |
| **Pulmonary Comorbidity** |  |  |  |
| No | --- | --- | --- |
| Asthma | 1.22 | 1.12, 1.32 | <0.001 |
| Cystic Fibrosis | 0.68 | 0.11, 2.26 | 0.599 |
| Pulmonary Fibrosis | 1.19 | 0.99, 1.41 | 0.064 |
| Bronchiectasis | 1.22 | 0.90, 1.60 | 0.180 |
| **Provider Type** |  |  |  |
| Advance Practice Provider | --- | --- | --- |
| Physician | 0.99 | 0.93, 1.04 | 0.595 |
| Trainee | 1.01 | 0.87, 1.17 | 0.866 |
| **Department Specialty** |  |  |  |
| Family Medicine | --- | --- | --- |
| Urgent Care | 1.27 | 1.19, 1.36 | <0.001 |
| Emergency Medicine | 1.65 | 1.55, 1.75 | <0.001 |
| **Visit Type** |  |  |  |
| In-Person | --- | --- | --- |
| Telehealth | 1.30 | 1.20, 1.42 | <0.001 |
| **Primary Diagnosis** |  |  |  |
| Bronchitis/Bronchiolitis | --- | --- | --- |
| Influenza | 0.76 | 0.68, 0.85 | <0.001 |
| Laryngitis/Pharyngitis | 0.93 | 0.81, 1.06 | 0.297 |
| Other | 1.12 | 0.52, 2.14 | 0.758 |
| Rhinitis | 0.26 | 0.22, 0.30 | <0.001 |
| Serous OM/Ear Disorders | 0.62 | 0.55, 0.70 | <0.001 |
| URI unspecified | 0.78 | 0.73, 0.84 | <0.001 |
| **Encounter Volume** |  |  |  |
| Low | --- | --- | --- |
| Mild | 0.98 | 0.89, 1.07 | 0.616 |
| Moderate | 1.17 | 1.07, 1.28 | <0.001 |
| High | 1.28 | 1.17, 1.40 | <0.001 |
| **Encounter Time** |  |  |  |
| Morning | --- | --- | --- |
| Afternoon | 1.05 | 1.00, 1.11 | 0.051 |
| **Charlson Group** |  |  |  |
| 0 | --- | --- | --- |
| 1-2 | 0.96 | 0.91, 1.02 | 0.206 |
| 3-4 | 1.00 | 0.90, 1.12 | 0.938 |
| 5+ | 1.20 | 1.09, 1.32 | <0.001 |
| **Antibiotic Prescribed** | 0.75 | 0.69, 0.82 | <0.001 |

Abbreviations: Oct, October; Mar, March; OM, otitis media; URI, upper respiratory infection.

**Supplementary eFigure 2: Relative Influence of Variables for Predicting 14-Day Repeat Respiratory-Related Healthcare Contact in the Post-Implementation Cohort**


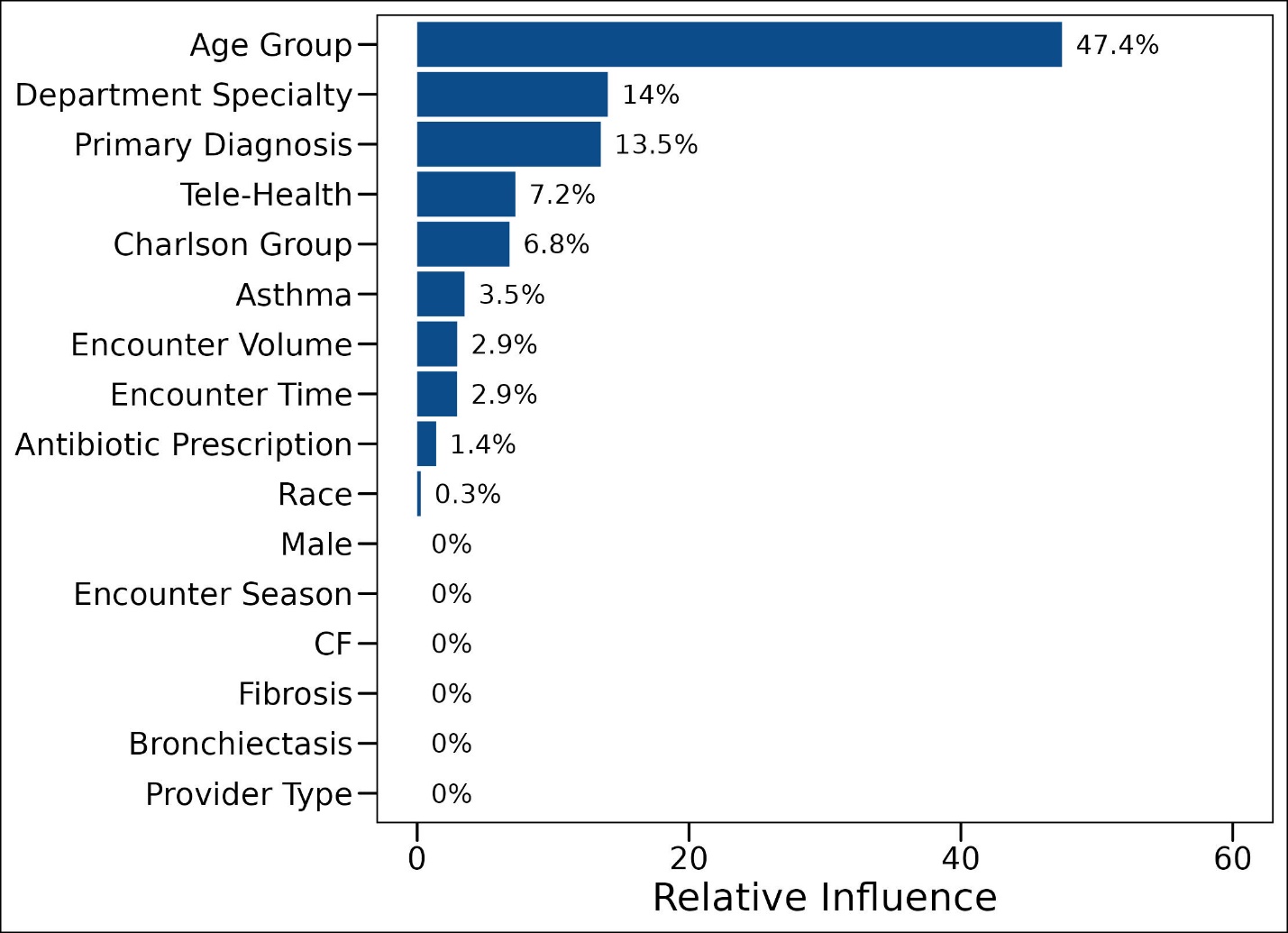


Abbreviations: CF, cystic fibrosis.

**EZ ID Respiratory Order Panel (Adult)**

**Quick links:**

- AskMayoExpert [hyperlink]
- Antimicrobial Quick Guide [hyperlink]
- Viral Rx Pad [hyperlink]

**Syndromes:**

- **Acute otitis media**
  - Medications
    - *Guidance text: For symptomatic management – use hyperlink below to print or consider adding smart phrase ViralRxAdult [108070] to the AVS.*
    - Antimicrobial therapy
      - *Guidance text: Default durations of therapy in this panel are 5 days. Typical duration of therapy in adult acute otitis media is 5-7 days for moderate symptoms and 10 days for severe (e.g., significant hearing loss, severe pain, and/or marked tympanic membrane erythema).*
      - No beta-lactam allergy
        - *Guidance text: Amoxicillin/clavulanate requires dosing adjustment in patients with renal dysfunction (i.e., CrCl <30 ml/min).*
        - Amoxicillin/clavulanate 875-125 mg PO bid x 5 days
      - Allergy to penicillin without immediate hypersensitivity reaction
        - *Guidance text: Cefdinir requires dosing adjustment in patients with renal dysfunction (i.e., CrCl <30 ml/min).*
        - Cefdinir 300 mg PO bid x 5 days
      - Allergy to both cephalosporins and penicillins with immediate hypersensitivity reactions
        - Doxycycline 100 mg PO bid x 5 days
    - Topical antimicrobial therapy
      - *Guidance text: Topical antibiotics may be added to oral antimicrobial regimens for select patients with tympanic membrane rupture.*
      - Ciprofloxacin-dexamethasone 0.3-0.1% otic suspension 4 drops into affected ear bid x 7 days
      - Ofloxacin 0.3% otic solution 10 drops into affected ear bid x 7 days
- **Acute Sinusitis**
  - *Guidance Text*
    - *Antimicrobial therapy is not indicated when symptoms of sinusitis have been present for < 10 days. These cases are typically reflective of a viral illness and should be managed with symptomatic management/supportive care.*
    - *Fewer than 2% of viral URIs progress to bacterial sinusitis. Most cases of bacterial sinusitis resolve without antibiotic treatment.*
    - *Antimicrobial treatment may be considered in patients with symptoms for > 10 days; patients with high fever, purulent nasal discharge, or facial pain for 3 or more days; or those with symptoms that have worsened after initial improvement.*
    - *Inappropriate antimicrobial prescribing may expose patients to unnecessary harm/risk such as C. difficile, adverse drug reactions, etc.*
  - Medications
    - *For Symptomatic Management - Use hyperlink below to print or consider adding smart phrase ViralRxAdult {108070} to the AVS.*
    - Antimicrobial therapy
      - No beta-lactam allergy
        - *Guidance text: Amoxicillin/clavulanate requires dosing adjustment in patients with renal dysfunction (i.e., CrCl <30 ml/min).*
        - Amoxicillin/clavulanate 875-125 mg PO bid x 5 days
      - Beta-lactam allergy
        - *Guidance text*

*Fluoroquinolones should be avoided unless other therapeutic alternatives are unavailable given risk for serious adverse effects.*

*Levofloxacin requires dosing adjustment in patients with renal dysfunction (i.e., CrCl <50 ml/min)*

- - - - - Doxycycline 100 mg PO bid x 5 days
        - Levofloxacin 500 mg PO daily x 5 days
- **Bronchitis (excluding COPD exacerbation)**
  - *Guidance text*
    - *Antibiotics are not indicated in the management of acute bronchitis and should not be prescribed in the absence of a diagnosed bacterial infection.*
    - *Inappropriate antimicrobial prescribing may expose patients to unnecessary harm/risk such as C. difficile, adverse drug reactions, etc.*
  - Medications
    - *Use hyperlink below to print or consider adding smart phrase ViralRxAdult {108070} to the AVS.*
    - Albuterol inhaler 1-2 puff every 6 hours prn wheezing (should be avoided in patients without underlying lung disease or wheezing)
    - Benzonatate 100 mg PO tid prn cough
- **Community Acquired Pneumonia**
  - Medications
    - Antimicrobial therapy
      - *Guidance text*
        - *Comorbidities to consider when selecting empiric therapy include chronic heart, lung, liver, or renal disease; diabetes mellitus; alcoholism; malignancy; asplenia; or recent use of other first line antibiotics.*
        - *Inpatient admission should be considered for patients with risk factors for MRSA or Pseudomonas aeruginosa, including prior respiratory isolation of either organism or receipt of parenteral antibiotics in the previous 90 days.*
        - *Macrolides – Avoid monotherapy with Azithromycin due to widespread resistance to Streptococcus pneumoniae (>25% at all Mayo Clinic sites)*
    - Patients without comorbidities
      - No beta-lactam allergy
        - *Guidance text: Amoxicillin requires dosing adjustment in patients with renal dysfunction (i.e., CrCl <30 ml/min).*
        - Amoxicillin 1000 mg PO tid x 5 days
      - Allergy to penicillin without immediate hypersensitivity reaction
        - *Guidance text: Cefdinir requires dosing adjustment in patients with renal dysfunction (i.e., CrCl <30 ml/min).*
        - Cefdinir 300 mg PO bid x 5 days
      - Allergy to both cephalosporins and penicillins with immediate hypersensitivity reactions
        - Doxycycline 100 mg PO bid x 5 days
    - Patients with comorbidities
      - No beta-lactam allergy
        - *Guidance text*

*Azithromycin may contribute to QT prolongation. Consider the use of beta-lactam/doxycycline combination in patients with known long QT, history of cardiac arrhythmias, or multiple current QT prolonging medications.*

*Amoxicillin/clavulanate requires dosing adjustment in patients with renal dysfunction (i.e., CrCl <30 ml/min).*

- - - - - Amoxicillin/clavulanate and azithromycin

Amoxicillin/clavulanate 875 mg-125 mg PO bid x 5 days

Azithromycin 500 mg PO x 1 followed by 250 mg PO x 4 days

- - - - - Amoxicillin/clavulanate and doxycycline

Amoxicillin/clavulanate 875 mg-125 mg PO bid x 5 days

Doxycycline 100 mg PO bid x 5 days

- - - - Allergy to penicillin without immediate hypersensitivity reaction
        - *Guidance text*

*Azithromycin may contribute to QT prolongation. Consider the use of beta-lactam/doxycycline combination in patients with known long QT, history of cardiac arrhythmias, or multiple current QT prolonging medications.*

*Cefdinir requires dosing adjustment in patients with renal dysfunction (i.e., CrCl <30 ml/min).*

- - - - - Cefdinir and azithromycin

Cefdinir 300 mg PO bid x 5 days

Azithromycin 500 mg PO x 1 followed by 250 mg PO x 4 days

- - - - - Cefdinir and doxycycline

Cefdinir 300 mg PO bid x 5 days

Doxycycline 100 mg PO bid x 5 days

- - - - Allergy to both cephalosporins and penicillins with immediate hypersensitivity reactions
        - *Guidance text: Levofloxacin requires dosing adjustment in patients with renal dysfunction (i.e., CrCl <50 ml/min)*
        - Levofloxacin 750 mg PO daily x 5 days
      - Recent use of first line drugs
        - *Guidance text: Levofloxacin requires dosing adjustment in patients with renal dysfunction (i.e., CrCl <50 ml/min)*
        - Levofloxacin 750 mg PO daily x 5 days
    - Symptomatic management
      - Albuterol inhaler 1-2 puff every 6 hours prn wheezing
      - Benzonatate 100 mg PO tid prn cough
  - Laboratory
    - *Guidance text: Refer to AskMayoExpert for current testing recommendations for influenza and COVID-19.*
    - Influenza A/B and RSV, PCR
- **COPD exacerbation**
  - *Guidance text: Cardinal symptoms of COPD include increased dyspnea, increased sputum volume, and increased sputum purulence. Treatment is indicated when all three symptoms are present, or with increased sputum purulence and one other symptom.*
  - Medications
    - Antimicrobial therapy
      - *Guidance text: Risk factors for Pseudomonas include: cystic fibrosis, bronchiectasis, known respiratory pseudomonas colonization, and IV antibiotics in previous 90 days.*
      - No beta-lactam allergy
        - *Guidance text: Amoxicillin/clavulanate requires dosing adjustment in patients with renal dysfunction (i.e. CrCl <30 ml/min).*
        - Amoxicillin/clavulanate 875-125 mg PO bid x 5 days
      - Allergy to penicillin without immediate hypersensitivity reaction
        - *Guidance text: Cefdinir requires dosing adjustment in patients with renal dysfunction (i.e., CrCl <30 ml/min).*
        - Cefdinir 300 mg PO bid x 5 days
        - Doxycycline 100 mg PO bid x 5 days
      - Allergy to both cephalosporins and penicillins with immediate hypersensitivity reaction
        - *Guidance text: Levofloxacin requires dosing adjustment in patients with renal dysfunction (i.e., CrCl <50 ml/min)*
        - Levofloxacin 750 mg PO daily x 5 days
        - Doxycycline 100 mg PO bid x 5 days
      - Risk factors for Pseudomonas
        - *Guidance text: Levofloxacin requires dosing adjustment in patients with renal dysfunction (i.e., CrCl <50 ml/min)*
        - Levofloxacin 750 mg PO daily x 5 days
    - Short acting bronchodilators
      - Albuterol 2.5 mg/3 mL nebulizer solution – inhale 3 mL by nebulization every 6 hours prn shortness of breath
      - Albuterol inhaler 2 puff every 4 hours prn shortness of breath
      - Ipratropium-albuterol 0.5-2.5 mg/3 mL nebulizer solution – inhale 3 mL four times daily prn shortness of breath
      - Ipratropium-albuterol 20-100 mcg/actuation inhaler – inhale 1 puff four times daily prn shortness of breath
    - Systemic steroids
      - Prednisone 40 mg PO daily x 5 days
- **Influenza**
  - Medications
    - *Guidance text*
      - *Treatment with antivirals is recommended for patients that are at high risk or have ongoing contact with high-risk individuals. Refer to AskMayoExpert for treatment recommendations.*
      - *Oseltamivir requires dosing adjustment in patients with renal dysfunction (i.e., CrCL <60 ml/min)*
    - Antimicrobial therapy
      - Oseltamivir 75 mg capsule (CrCl >60 ml/min)
        - Oseltamivir 75 mg PO bid x 5 days
      - Oseltamivir 30 mg capsule (CrCl 31-60 ml/min)
        - Oseltamivir 30 mg PO bid x 5 days
      - Oseltamivir 30 mg capsule (CrCl 11-31 ml/min)
        - Oseltamivir 30 mg PO daily x 5 days
    - Symptomatic management
      - *Guidance text: For Symptomatic Management - Use hyperlink below to print or consider adding smart phrase ViralRxAdult {108070} to the AVS.*
      - Albuterol inhaler 1-2 puff every 6 hours prn wheezing
      - Benzonatate 100 mg PO tid prn cough
  - Laboratory
    - *Guidance text: Refer to AskMayoExpert for current testing recommendations for influenza and COVID-19.*
    - Influenza A/B and RSV, PCR
- **Pharyngitis**
  - *Guidance text*
    - *Pharyngitis is commonly caused by viruses. Antibiotic treatment is only indicated for streptococcal pharyngitis.*
    - *Inappropriate antimicrobial prescribing may expose patients to unnecessary harm/risk such as C. difficile, adverse drug reactions, etc.*
    - *Features suggesting non-streptococcal (viral) pharyngitis, especially when present in combination: conjunctivitis, rhinorrhea, coryza, cough, hoarseness, discrete ulcerative stomatitis, viral exanthema, splenomegaly, or non-scarlatiniform rash*
  - Medications
    - *Guidance text*
      - *Suspected streptococcal pharyngitis should not be treated unless confirmed by testing*
      - *For symptomatic management – use hyperlink below to print or consider adding smart phrase ViralRxAdult {108070} to the AVS.*
    - Antimicrobial therapy
      - No beta-lactam allergy
        - *Guidance text: Amoxicillin requires dosing adjustment in patients with renal dysfunction (i.e. CrCl <30 ml/min).*
        - Penicillin VK 500 mg PO bid x 10 days
        - Amoxicillin 500 mg PO bid x 10 days
        - Alternative dosing strategy: Amoxicillin 1000 mg daily for 10 days
      - Allergy to penicillin without immediate hypersensitivity reaction
        - Cephalexin 500 mg PO bid x 10 days
        - Cefadroxil 500 mg PO bid x 10 days
      - Allergy to both cephalosporins and penicillins with immediate hypersensitivity reactions
        - Azithromycin 500 mg PO x 1 followed by 250 mg PO daily x 4 days
        - Alternative dosing strategy: Azithromycin 500 mg PO daily x 3 days
      - Clinic administered medications
        - Penicillin benzathine 1.2 million units IM x 1 dose
  - Laboratory
    - *Guidance text*
      - *Patients age less than 3 years – Routine testing not recommended. Test/Treat only if patients have fever AND pharyngitis AND household contact with known streptococcal pharyngitis in the past 7 days.*
      - *Patients age 3 years and older – Use Centor criteria*
      - *A score of 3 or more generally warrants testing. Testing is not usually indicated for a score of 2 or less.*
    - Streptococcus Group A, molecular detection, PCR, throat

**EZ ID Respiratory Order Panel (Pediatric)**

**Quick links:**

- AskMayoExpert [hyperlink]
- Antimicrobial Quick Guide [hyperlink]
- Viral Rx Pad [hyperlink]

**Syndromes:**

- **Acute otitis media**
  - *Guidance text*
    - *Consider observation in the absence of severe symptoms (high fever, moderate/severe otalgia, otalgia > 48 hours, or toxic appearance):*
      - *Children 6-23 months with unilateral acute otitis media*
      - *Children age 2 or older, regardless of laterality*
  - Medications
    - Antimicrobial therapy
      - *Guidance text*
        - Antibiotic durations for amoxicillin, amoxicillin-clavulanate, *cefdinir:*

*5 days: Patients 6 years of age or older*

*7 days: Patients 2 years of age to less than 6 years of age*

*10 days: Patients less than 2 years of age or patients greater than 2 years of age with severe symptoms, chronic acute otitis media, recurrent acute otitis media, or tympanic membrane perforation*

- - - - No beta-lactam allergy
        - *Guidance text*

*Recommended amoxicillin dosing 90 mg/kg/day. Choose preferred formulation*

*Recommended amoxicillin-pot clavulanate tablets dosing 45 mg/kg/dose (max dose 1312 mg).*

*Amoxicillin requires dosing adjustment in patients with renal dysfunction (i.e. CrCl <30 ml/min)*

*Amoxicillin/clavulanate requires dosing adjustment in patients with renal dysfunction (i.e. CrCl <30 ml/min)*

- - - - - Amoxicillin 400 mg/5 mL suspension – 45 mg/kg PO bid
        - Amoxicillin 500 mg capsules – 45 mg/kg PO bid
        - Amoxicillin/clavulanate 600-42.9 mg/5 mL suspension – 45 mg/kg PO bid
        - Amoxicillin/clavulanate 875-125 mg tablet (patient weight >17 kg) – 45 mg/kg PO bid
      - Allergy to penicillin without immediate hypersensitivity reaction
        - *Guidance text*

*Recommended dosing 7 mg/kg (max dose 300 mg). Choose preferred formulation.*

*Cefdinir requires dosing adjustment in patients with renal dysfunction (i.e. CrCl <30 ml/min).*

- - - - - Cefdinir 250 mg/5 mL suspension – 7 mg/kg PO bid
        - Cefdinir 300 mg capsule (patient weight >38 kg) – 7 mg/kg PO bid
      - Allergy to both cephalosporins and penicillins with immediate hypersensitivity reactions
        - *Guidance text*

*Macrolides - Avoid use unless no other reasonable options exist because of widespread resistance in Streptococcus pneumoniae and potential for treatment failure.*

*Recommended azithromycin dosing 10 mg/kg then 5 mg/kg (max dose 500 mg then 250 mg). Choose preferred formulation.*

- - - - - Azithromycin 200 mg/ 5 mL suspension – 10 mg/kg PO x 1 then 5 mg/kg PO daily x 4 days
        - Azithromycin 250 mg tablets – 10 mg/kg PO x 1 then 5 mg/kg PO daily x 4 days
      - Topical Antimicrobial Therapy
        - *Guidance text: Topical antibiotics may be used in patients with tympanostomy tubes or added to oral antimicrobial regimens for select patients > 6 months of age with tympanic membrane rupture.*
        - Ciprofloxacin-dexamethasone 0.3-0.1% otic suspension 4 drops into affected ear bid x 7 days
        - Ofloxacin 0.3% otic solution 10 drops into affected ear bid x 7 days
      - Clinic administered medications
        - *Guidance text: Recommended ceftriaxone dosing 50 mg/kg (max dose 1000 mg). Choose preferred formulation.*
        - Ceftriaxone injection in lidocaine 1% - 50 mg/kg IM once
        - Ceftriaxone injection – 50 mg/kg IM once
    - Symptomatic management
      - *Guidance text: Use hyperlink below to print or consider adding smart phrase ViralRxPediatric {108071} to the AVS.*
      - Lidocaine viscous 2% mucosal solution – Use only when ear drum is intact.
        - Lidocaine 2% mucosal solution – instill 3-5 drops into affected ear(s) eery 2 hours prn (max 5 doses (25 drops) in each ear in a 24-hour period)
- **Acute sinusitis**
  - *Guidance text*
    - *Antimicrobial therapy is not indicated when symptoms of sinusitis have been present for < 10 days. These cases are typically reflective of a viral illness and should be managed with symptomatic management/supportive care.*
    - *Fewer than 2% of viral URIs progress to bacterial sinusitis. Most cases of bacterial sinusitis resolve without antibiotic treatment.*
    - *Antimicrobial treatment may be considered in patients with symptoms for > 10 days; patients with high fever, purulent nasal discharge, or facial pain for 3 or more days; or those with symptoms that have worsened after initial improvement.*
    - *Inappropriate antimicrobial prescribing exposes patients to unnecessary harm/risk such as C. difficile, adverse drug reactions, etc.*
  - Medications
    - *Guidance text: For symptomatic management, use hyperlink below to print or consider adding smart phrase ViralRxPediatric {108071} to the AVS.*
    - Antimicrobial therapy
      - No beta-lactam allergy
        - *Guidance text*

*Recommended suspension dosing 90 mg/kg/day (max dose 4000 mg/day).*

*Recommended tablet dosing 90 mg/kg/day (max dose 2625 mg). Choose preferred formulation.*

*Amoxicillin/clavulanate requires dosing adjustment in patients with renal dysfunction (i.e. CrCl <30 ml/min)*

- - - - - Amoxicillin/clavulanate 600-42.9 mg/5 mL suspension – 45 mg/kg PO bid x 7 days
        - Amoxicillin/clavulanate 875-125 mg tablets (patient weight >17 kg) – 45 mg/kg PO bid x 7 days
      - Allergy to penicillin without immediate hypersensitivity reaction
        - *Guidance text*

*Recommended dosing 7 mg/kg (max dose 300 mg). Choose preferred formulation.*

*Cefdinir requires dosing adjustment in patients with renal dysfunction (i.e. CrCl <30 ml/min)*

- - - - - Cefdinir 250 mg/5 mL suspension – 7 mg/kg PO bid x 7 days
        - Cefdinir 300 mg capsule (patient weight >38 kg) – 7 mg/kg PO bid x 7 days
      - Allergy to both cephalosporins and penicillins with immediate hypersensitivity reactions
        - *Guidance text*

*Fluoroquinolones should be avoided unless other therapeutic alternatives are unavailable given risk for serious adverse effects, especially in patients <12 years old.*

*Recommended dosing 10 mg/kg (max dose 500 mg). Choose preferred formulation.*

*Levofloxacin requires dosing adjustment in patients with renal dysfunction (i.e. CrCl <30 ml/min)*

- - - - - Levofloxacin 500 mg tablet (patient weight >22 kg) – 10 mg/kg PO daily x 7 days
      - Clinic administered medications
        - *Guidance text: Recommended ceftriaxone dosing 50 mg/kg (max dose 1000 mg). Choose preferred formulation.*
        - Ceftriaxone injection in lidocaine 1% - 50 mg/kg IM once
        - Ceftriaxone injection – 50 mg/kg IM once
- **Bronchiolitis**
  - *Guidance text*
    - *Antibiotics are not indicated in the management of acute bronchitis/bronchiolitis and should not be prescribed in the absence of a diagnosed bacterial infection.*
    - *Inappropriate antimicrobial prescribing exposes patients to unnecessary harm/risk such as C. difficile, adverse drug reactions, etc.*
    - *Use hyperlink below to print or consider adding smart phrase ViralRxPediatric {108071} to the AVS.*
    - *Short acting bronchodilators are NOT recommended in patients with bronchiolitis*
- **Bronchitis**
  - *Guidance text*
    - *Antibiotics are not indicated in the management of acute bronchitis/bronchiolitis and should not be prescribed in the absence of a diagnosed bacterial infection.*
    - *Inappropriate antimicrobial prescribing exposes patients to unnecessary harm/risk such as C. difficile, adverse drug reactions, etc.*
  - Medications – Symptomatic management
    - *Guidance text: Use hyperlink below to print or consider adding smart phrase ViralRxPediatric {108071} to the AVS.*
    - Albuterol inhaler – should be avoided in patients without underlying lung disease or wheezing
      - Albuterol 90 mcg/actuation inhaler - Inhale 2 puffs every 4 hours prn shortness of breath
    - Inhalational spacing device – spacer
      - Inhalational spacer – 1 devise prn use with inhaler
    - Albuterol 1.25 mg/3 mL nebulizer solution – inhale 3 mL (1.25 mg total) by nebulization every 4 hours prn shortness of breath
- **Community acquired pneumonia**
  - *Guidance text*
    - *Inpatient management is indicated in children and infants with one or more of the following:*
      - *Patients who are <6 months of age with suspected bacterial CAP, fever, or hypoxemia (O2 sat <90% in room air)*
      - *Moderate/severe CAP defined as respiratory distress (tachycardia, dyspnea, retractions, nasal flaring, altered mental status, grunting, apnea) and hypoxemia (O2 sat <90% in room air)*
      - *Suspected or documented CAP caused by a pathogen with increased virulence (i.e. CA-MRSA)*
      - *Concern about home observation, ability to follow up, or are unable to comply with therapy*
  - Medications
    - Antimicrobial therapy (Children 5-18 years)
      - *Guidance text*
        - *Presumed bacterial pneumonia includes high fever, focal crackles, and/or focal infiltrate on chest radiography.*
        - *Presumed atypical pneumonia includes low-grade fever, scattered crackles, and/or interstitial infiltrate on chest radiography.*
        - *If presenting with mixed features of both bacterial and atypical pneumonia, use a combination of antimicrobial agents from both criteria.*
      - Presumed BACTERIAL pneumonia
        - No beta-lactam allergy

*Guidance text*

*Recommended dosing 90 mg/kg/day (max dose 4000 mg/day). Choose preferred formulation*

*Amoxicillin requires dosing adjustment in patients with renal dysfunction (i.e. CrCl < 30 ml/min)*

Amoxicillin 400mg/5 ml - 45mg/kg/dose BID (max 4g/day)

Amoxicillin 250 mg chewable - 45mg/kg/dose BID (max 4g/day)

Amoxicillin 500 mg capsule - 45mg/kg/dose BID (max 4g/day)

- - - - - Allergy to penicillin without immediate hypersensitivity reaction

*Guidance text*

*Recommended dosing 7 mg/kg (max dose 300 mg): 300 mg (max dose value) Choose preferred formulation.*

*Cefdinir requires dosing adjustment in patients with renal dysfunction (i.e. CrCl <30 ml/min)*

Cefdinir 250mg/5mL suspension - 7 mg/kg/dose BID x 7 days

Cefdinir 300 mg capsules (Patient weight > 38 kg) - 7 mg/kg/dose BID x 7 days (maximum 600 mg/day)

- - - - - Allergy to both cephalosporins and penicillins with immediate hypersensitivity reactions

*Guidance text*

*Fluoroquinolones should be avoided unless other therapeutic alternatives are unavailable given risk for serious adverse effects, especially in patients <12 years old.*

*Recommended doxycycline dosing 2 mg/kg (max dose 100 mg): 100 mg (max dose value) Choose preferred formulation.*

*Recommended levoFLOXacin dosing 10 mg/kg (max dose 750 mg): 577 mg (actual weight) Choose preferred formulation.*

*Levofloxacin requires dosing adjustment in patients with renal dysfunction (i.e. CrCl <30 ml/min)*

Doxycycline

Doxycycline

Levofloxacin solution 25may be used in children > 12 years but should be reserved for cases where other therapeutic alternatives are unavailable: Levofloxacin 25 mg/mL solution- 10 mg/kg/dose QD x7 days (maximum 750mg/day)

Levofloxacin oral tablet (patient weight > 40 kg) may be used in children > 12 years but should be reserved for cases where other therapeutic alternatives are unavailable: Levofloxacin 500 mg tablet -10 mg/kg/dose QD x7 days (maximum 750mg/day)

- - - - Presumed ATYPICAL pneumonia
        - *Guidance text*

*Recommended azithromycin dosing 10 mg/kg then 5 mg/kg (max dose 500 mg then 250 mg): 500 mg (max dose value) then 250 mg (max dose value). Choose preferred formulation.*

*Recommended doxycycline dosing 2 mg/kg (max dose 100 mg): 100 mg (max dose value). Choose preferred formulation.*

- - - - - Azithromycin 200 mg/5mL - 10 mg/kg (maximum 500 mg/dose) on day 1 followed by 5 mg/kg/dose (maximum 250 mg/dose) once daily on days 2 through 5.
        - Azithromycin 250 mg tabs - 10 mg/kg (maximum 500 mg/dose) on day 1 followed by 5 mg/kg/dose (maximum 250 mg/dose) once daily on days 2 through 5.
        - Doxycycline monohydrate 25 mg/5mL suspension - 2 mg/kg/dose BID (maximum 100 mg PO BID) x 7 days
        - Doxycycline monohydrate 100 mg capsules (patients weighing greater than 40 kg) - 2 mg/kg/dose BID (maximum 100 mg PO BID) x 7 days
    - Clinic administered medications (parenteral)
      - *Guidance text: Recommended ceftriaxone dosing 50 mg/kg (max dose 1000 mg). Choose preferred formulation.*
      - Ceftriaxone injection in lidocaine 1% - 50 mg/kg IM once
      - Ceftriaxone injection – 50 mg/kg IM once
    - Symptomatic management
      - *Guidance text: Use hyperlink below to print or consider adding smart phrase ViralRxPediatric {108071} to the AVS.*
      - Albuterol inhaler – should be avoided in patients without underlying lung disease or wheezing
        - Albuterol 90 mcg/actuation inhaler - Inhale 2 puffs every 4 hours prn shortness of breath
      - Inhalational spacing device – spacer
        - Inhalational spacer – 1 devise prn use with inhaler
      - Albuterol 1.25 mg/3 mL nebulizer solution – inhale 3 mL (1.25 mg total) by nebulization every 4 hours prn shortness of breath
  - Laboratory
    - *Guidance text: Refer to AskMayoExpert for current testing recommendations for influenza and COVID-19.*
    - Influenza A/B and RSV, PCR
- **Influenza**
  - Medications
    - Antiviral therapy
      - *Guidance text: Oseltamivir requires dosing adjustment in patients with renal dysfunction (i.e. CrCl <60 ml/min).*
      - 12 months and older
        - Weight ≤15 kg

Oseltamivir 6 mg/mL suspension – 30 mg PO bid x 5 days

- - - - - Weight >15-23 kg

Oseltamivir 6 mg/mL suspension – 45 mg PO bid x 5 days

- - - - - Weight >23-40 kg

Oseltamivir 6 mg/mL suspension – 60 mg PO bid x 5 days

- - - - - Weight >40 kg (suspension)

Oseltamivir 6 mg/mL suspension – 75 mg PO bid x 5 days

- - - - - Weight >40 kg (capsule)

Oseltamivir 75 mg PO bid x 5 days

- - - Symptomatic management
      - *Guidance text: Use hyperlink below to print or consider adding smart phrase ViralRxPediatric {108071} to the AVS.*
      - Albuterol inhaler – should be avoided in patients without underlying lung disease or wheezing
        - Albuterol 90 mcg/actuation inhaler - Inhale 2 puffs every 4 hours prn shortness of breath
      - Inhalational spacing device – spacer
        - Inhalational spacer – 1 devise prn use with inhaler
      - Albuterol 1.25 mg/3 mL nebulizer solution – inhale 3 mL (1.25 mg total) by nebulization every 4 hours prn shortness of breath
  - Laboratory
    - *Guidance text: Refer to AskMayoExpert for current testing recommendations for influenza and COVID-19.*
    - Influenza A/B and RSV, PCR
    - SARS Coronavirus-2, COVID-19, PCR
- **Pharyngitis**
  - *Guidance text*
    - *Pharyngitis is commonly caused by viruses. Antibiotic treatment is only indicated for streptococcal pharyngitis. Inappropriate antimicrobial prescribing exposes patients to unnecessary harm/risk such as C. difficile, adverse drug reactions, etc.*
    - *Features suggesting non-streptococcal (viral) pharyngitis, especially when present in combination: conjunctivitis, rhinorrhea, coryza, cough, hoarseness, discrete ulcerative stomatitis, viral exanthema, splenomegaly, or non-scarlatiniform rash*
  - Medications
    - *Guidance text: For symptomatic management, use hyperlink below to print or consider adding smart phrase ViralRxPediatric {108071} to the AVS.*
    - Antimicrobial therapy
      - *Guidance text: Suspected streptococcal pharyngitis should not be treated unless confirmed by testing.*
      - No beta-lactam allergy
        - Penicillin VK

Weight less or equal to 27 kg – solution

Penicillin V potassium 250 mg/5 mL solution – 250 mg PO bid x 10 days

Weight greater than 27 kg – solution

Penicillin V potassium 250 mg/ 5 mL solution – 500 mg PO bid x 10 days

Weight greater than 27 kg – tablet

Penicillin V potassium 500 mg tablet – 1 tablet PO bid x 10 days

- - - - - Amoxicillin

*Guidance text*

*Recommended dosing 25 mg/kg (max dose 500 mg). Choose preferred formulation.*

*Amoxicillin requires dosing adjustment in patients with renal dysfunction (i.e. CrCl <30 ml/min).*

400 mg/5 mL suspension

Amoxicillin 400 mg/5 mL suspension – 25 mg/kg PO bid x 10 days

Weight greater than 20 kg – capsule

Amoxicillin 500 mg PO bid x 10 days

- - - - Allergy to penicillin without immediate hypersensitivity reaction
        - *Guidance text*

*Recommended dosing 20 mg/kg (max dose 500 mg). Choose preferred formulation.*

*Cephalexin requires dosing adjustment in patients with renal dysfunction (i.e. CrCl <50 ml/min)*

- - - - - Cephalexin 250 mg/5 mL suspension – 20 mg/kg PO bid x 10 days
        - Cephalexin 500 mg capsule (patient weight >20 kg) – 1 capsule PO bid x 10 days
      - Allergy to both cephalosporins and penicillins with immediate hypersensitivity reactions
        - *Guidance text*

*Macrolides - Avoid use unless no other reasonable options exist because of widespread resistance in Streptococcus pneumoniae and potential for treatment failure.*

*Recommended azithromycin dosing 12 mg/kg then 6 mg/kg (max dose 500 mg). Choose preferred formulation.*

- - - - - Azithromycin 200 mg/5 mL suspension – 12 mg/kg PO x 1 followed by 6 mg/kg daily x 4 days
        - Azithromycin 250 mg tablet – 12 mg/kg PO x 1 followed by 6 mg/kg daily x 4 days
      - Clinic administered medications
        - Penicillin G benzathine (patient weight <27 kg) – 600,000 units IM once
        - Penicillin G benzathine (patient weight >27 kg) – 1.2 million units IM once
  - Laboratory
    - *Guidance text*
      - *Patients age less than 3 years – Routine testing not recommended. Test/Treat only if patients have fever AND pharyngitis AND household contact with known streptococcal pharyngitis in the past 7 days.*
      - *Patients age 3 years and older – Use Centor criteria.*
      - *A score of 3 or more generally warrants testing. Testing is not usually indicated for a score of 2 or less.*
    - Group A Streptococcus PCR, throat
